# Exploring the Lean Phenotype of Glutathione-Depleted Mice: Thiol, Amino Acid and Fatty Acid Profiles

**DOI:** 10.1371/journal.pone.0163214

**Published:** 2016-10-27

**Authors:** Amany K. Elshorbagy, Fredrik Jernerén, Cheryl L. Scudamore, Fiona McMurray, Heather Cater, Tertius Hough, Roger Cox, Helga Refsum

**Affiliations:** 1 Department of Physiology, Faculty of Medicine, University of Alexandria, Alexandria, Egypt; 2 Department of Pharmacology, University of Oxford, Oxford, United Kingdom; 3 Mary Lyon Centre, MRC Harwell Institute, Harwell Campus, Oxford, United Kingdom; 4 MRC Harwell Institute, Mammalian Genetics Unit, Harwell Campus, Oxford, United Kingdom; 5 Institute of Basic Medical Sciences, Department of Nutrition, University of Oslo, Oslo, Norway; Hospital Infantil Universitario Nino Jesus, SPAIN

## Abstract

**Background:**

Although reduced glutathione (rGSH) is decreased in obese mice and humans, block of GSH synthesis by buthionine sulfoximine (BSO) results in a lean, insulin-sensitive phenotype. Data is lacking about the effect of BSO on GSH precursors, cysteine and glutamate. Plasma total cysteine (tCys) is positively associated with stearoyl-coenzyme A desaturase (SCD) activity and adiposity in humans and animal models.

**Objective:**

To explore the phenotype, amino acid and fatty acid profiles in BSO-treated mice.

**Design:**

Male C3H/HeH mice aged 11 weeks were fed a high-fat diet with or without BSO in drinking water (30 mmol/L) for 8 weeks. Amino acid and fatty acid changes were assessed, as well as food consumption, energy expenditure, locomotor activity, body composition and liver vacuolation (steatosis).

**Results:**

Despite higher food intake, BSO decreased particularly fat mass but also lean mass (both P<0.001), and prevented fatty liver vacuolation. Physical activity increased during the dark phase. BSO decreased plasma free fatty acids and enhanced insulin sensitivity. BSO did not alter liver rGSH, but decreased plasma total GSH (tGSH) and rGSH (by ~70%), and liver tGSH (by 82%). Glutamate accumulated in plasma and liver. Urine excretion of cysteine and its precursors was increased by BSO. tCys, rCys and cystine decreased in plasma (by 23–45%, P<0.001 for all), but were maintained in liver, at the expense of decreased taurine. Free and total plasma concentrations of the SCD products, oleic and palmitoleic acids were decreased (by 27–38%, P <0.001 for all).

**Conclusion:**

Counterintuitively, block of GSH synthesis decreases circulating tCys, raising the question of whether the BSO-induced obesity-resistance is linked to cysteine depletion. Cysteine-supplementation of BSO-treated mice is warranted to dissect the effects of cysteine and GSH depletion on energy metabolism.

## Introduction

A link between obesity and oxidative stress has long been recognized. Markers of protein oxidation and lipid peroxidation increase in obesity, and often improve with weight loss interventions [1]. Plasma concentration of the antioxidant glutathione (GSH) decreases in obesity [2], and the ratio of disulfide/reduced sulfhydryl (GSSG/rGSH) increases [3]. Plasma total GSH (tGSH) correlates inversely with BMI and fat mass [4, 5]. In obese individuals, adipose tissue tGSH was ~30% lower than in lean subjects [6], and muscle rGSH/GSSG was decreased [7]. Data *in vitro* suggests that oxidative stress may be a causal factor in obesity. Decreased GSH accelerated adipogenesis in 3T3-L1 cells, and enhanced preadipocyte differentiation and lipid droplet formation [8].

Oxidative stress also links obesity to obesity-related morbidity. Oxidative stress preceded the development of insulin resistance following high-fat feeding in mice [9]. High-fat feeding in lean men lowered muscle rGSH/GSSG, increased ROS emission and impaired insulin sensitivity [7]. In rats, diet-induced fatty liver was linked to decreases in liver GSH and antioxidant capacity, and was prevented by GSH-restoring therapy [10].

In apparent conflict with data showing GSH depletion in obesity, insulin resistance and fatty liver, two models in which GSH synthesis is blocked feature leanness, hypermetabolism and insulin sensitivity. These are mice in which glutamate cysteine ligase (GCL), the rate-limiting GSH synthesis enzyme, is inhibited by a genetic defect (GCL-modifier subunit knockout, *Gclm*^*-/-*^) [11, 12] or by buthionine sulfoximine (BSO) [13, 14]. *Gclm*^*-/-*^ mice also exhibit protection against fatty liver, and hepatic suppression of the lipogenic enzyme stearoyl CoA desaturase-1 (SCD1) [11, 12], but these features have not been tested in BSO-treated mice. As proposed by Kendig *et al*, GSH depletion is unlikely to be the cause of obesity-resistance conferred by GCL inhibition [11]. This raises the question of whether the lean phenotype of *Gclm*^*-/-*^ and BSO-treated mice is in fact linked to decreased cysteine.

Cysteine is a rate-limiting precursor in GSH synthesis. Plasma total cysteine (tCys) is consistently elevated in obese humans [5, 15] and correlates with fat mass [16]. Several transgenic and dietary rodent models in which tCys levels decrease are lean [17, 18]. As a GSH precursor, cysteine might be expected to accumulate with GCL inhibition. However, GSH acts as a cysteine reservoir that supplies cysteine extracellularly when acted upon by gamma-glutamyltransferase (GGT) [19]. (The gamma-glutamyl cycle and the metabolic relationship among the sulfur amino acids are depicted in S1 Fig in the Supporting Information). *Gclm*^*-/-*^ mice indeed paradoxically feature decreased plasma free cysteine and cystine [11]. Yet most tCys in plasma is protein-bound. It is not known if this quantitatively major form decreases with GCL inhibition. To further explore the link between thiols and energy metabolism, we characterized BSO-treated mice in terms of thiol, amino acid and fatty acid profiles, SCD changes and liver steatosis.

## Methods

### Animal Husbandry

Mice were kept and studied in accordance with UK Home Office legislation (Animal (Scientific Procedures) Act 1986 Amendment Regulations 2012 (SI 4 2012/3039).) and local ethical guidelines issued by the Medical Research Council (Responsibility in the Use of Animals for Medical Research, July 1993; home office license 30/3146). All effort was made to reduce the number of animals used and to refine both procedures and husbandry. Cohort sizes were selected using power calculations using data from pilot studies. Projects were approved and reviewed by the Harwell animal welfare and ethical review board (AWERB).

Mice were kept under controlled light (12 hr light and 12 hr dark cycle, dark 7 pm-7 am), temperature (21 ± 2°C) and humidity (55% ± 10%) conditions. They had free access to water (10 ppm chlorine) and, prior to start of the experiment, were fed a commercial diet (SDS Rat and Mouse No.3 Breeding diet (RM3)) containing 3.36 gm% fat, 22.45 gm% protein and 71.21 gm% carbohydrate. Where applicable, phenotyping tests were performed according to IMPReSS (International Phenotyping Resource for Standardised Screens) standardized protocols as described at https://www.mousephenotype.org/impress. Unless otherwise stated, phenotyping tests are reported with reference to the number of weeks after start of BSO administration.

### High-fat diet and BSO administration

Male C3H/HeH mice were maintained on the RM3 diet from weaning till maturity. At 11 weeks of age, mice were shifted to a high-fat diet (HFD) with or without BSO in drinking water at a concentration of 30 mmol/L. The HFD (D12451, Open Source Diets) provided 45% calories from fat, 20% from protein and 35% from carbohydrates. After 8 weeks, mice were anaesthetised using isoflurane (after an overnight fast) and blood was collected from the retro-orbital sinus. The mice were then culled by exsanguination and the liver harvested for biochemical and histological analysis.

### Body Composition

Body mass was measured at baseline and weekly thereafter on scales calibrated to 0.01 g. Analysis of body composition was performed at baseline, and every two weeks thereafter by an Echo MRI whole body composition analyzer (Echo Medical System, Houston, TX). Terminal dissection and measurement of mesenteric (peri-intestinal) fat weight was also performed to assess abdominal adiposity.

### Energy expenditure, physical activity and food and water consumption

Two weeks after the start of BSO treatment, mice were individually housed in PhenoMaster cages (TSE Systems, Bad Homburg, Germany) for collection of energy intake/expenditure related data over 24 hours. The cage system includes weighing sensors that measure the amount of food and liquid consumed over time, and a photobeam-based activity monitoring system that records ambulatory movements in the horizontal and vertical planes. An indirect gas calorimetry system simultaneously measured oxygen consumption (VO_2_), carbon dioxide production (VCO_2_) and respiratory exchange ratio (RER). VO_2_ and VCO_2_ data was normalized to lean body mass measured in the same week by EchoMRI.

### Evaluation of insulin sensitivity at week 6

At week 6, mice were fasted for 6 hours during the light phase, and a blood sample collected by a tail bleed in EDTA-treated tubes for measurement of plasma glucose, insulin and leptin. Plasma insulin and leptin levels were measured using a mouse endocrine MILLIPLEX kit (MILLIPLEX MAP, Millipore) and a Bio-Plex 200 system (Bio-Rad) according to the manufacturer's instructions. The homeostasis model assessment of insulin resistance index (HOMA-IR) calculated as: [plasma insulin (ng/mL) × plasma glucose (mg/dL)/405], was used as a measure of insulin sensitivity. Six-hour fasting has been recommended for estimation of HOMA-IR in mice [20, 21].

### Terminal plasma and liver assays

Terminal lithium-heparin plasma samples from overnight-fasted mice were used for measurement of amino acids, fatty acids, thiol redox profile and clinical biochemistry parameters as detailed below.

### Clinical biochemistry

Plasma was analysed for glucose, creatinine, alanine aminotransferase (ALT), total protein, albumin, triglycerides, total cholesterol, HDL cholesterol, and LDL cholesterol on board a Beckman Coulter AU680 clinical chemistry analyser using reagents and settings recommended by the manufacturer.

Plasma GGT activity was assayed by a calorimetric kit (BioVision, California, US, Catalogue number K784-100) according to the manufacturer’s instructions.

### Amino acid analysis

For the amino thiols, the prefix “r” is used to denote the reduced form (e.g. rCys), while the prefix “t” indicates the total plasma concentration (e.g. tHcy, comprising the sum of free reduced, free homogeneous and mixed disulphide, and protein-bound forms of homocysteine).

Amino acids were assayed by liquid chromatography-tandem mass spectrometry (LC-MS/MS) using a Prominence LC-20AD_XR_ binary pump (Shimadzu, Kyoto, Japan) coupled to a QTRAP 5500 hybrid triple quadropole mass spectrometer (AB Sciex, Framingham, MA, US). Plasma methionine, tCys, total homocysteine (tHcy), cystathionine, and tGSH were analysed in a single run [22]. The protocol [22] was modified to include SAM, SAH, arginine, valine, proline, leucine, isoleucine, phenylalanine, tyrosine, ornithine, and tryptophan. Plasma taurine, glutamic acid, serine, and glutamine were analysed in a separate run using a modification of a previously described method [23]. Quantitation was based on comparisons with standard curves corrected for the presence of isotopically labelled internal standards. Reduced cysteine (rCys), cystine and rGSH were analysed in plasma stabilized with 3 volumes of 4% perchloric acid (PCA), using the same chromatographic conditions described above. Coefficients of variation (CV) for all amino acid analyses were ≤ 5%, except for SAM, SAH, taurine, tryptophan, GSH and cystathionine, which were < 10%.

Liver amino acid concentrations were measured after homogenisation of liver tissue by sonication in 5 volumes of water. The homogenate was processed and analysed as per plasma methods.

### Fatty acid analysis

Total plasma fatty acids were analysed using gas-chromatography coupled to mass spectrometry on a Focus GC-DSQ II (Thermo Scientific, Waltham, MA, US) described [24]. Plasma free fatty acids were analysed by LC-MS/MS as described previously [25], with minor modifications. %CV for total 14:0, 18:3n-3, 20:5n-3, free16:0, and both total and free 20:3n-6, and 22:6n-3 were ≤ 10%. The %CV for total 12:0 and free18:0 and 20:4n-6 were ≤ 15%. %CV for all for other total and free fatty acids were ≤ 5%.

### Stearoyl coenzyme A desaturase activity indices

Desaturase activity indices were estimated from total plasma fatty acids and free fatty acid concentrations, as product/precursor ratios [26]. SCD1-16 activity was calculated as 16:1n-7/16:0, SCD1-18 activity as 18:1n-9/18:0.

### Urine measurements

Total glutathione and sulfur amino acids were measured in urine by LC-MS/MS using a similar method to that described above for plasma. The measurements were normalized to creatinine (assayed on a Beckman Coulter AU680 clinical chemistry analyser using reagents and settings recommended by the manufacturer).

### Liver pathology

Liver tissue was harvested for pathological evaluation, fixed in 10% neutral buffered formalin and processed routinely to generate haematoxylin and eosin-stained tissue sections. Tissue sections (N = 5 per group) were evaluated microscopically, and liver vacuolation was scored semi-quantitatively, from 0 –no vacuolation present to 5 –all hepatocytes contain vacuoles [27].

### Statistical analysis

Serial body composition measurements, as well as urine analytes, are presented as mean ± SEM, and compared by repeated measures ANOVA. Physical activity counts, and food and water consumption are also presented as mean ± SEM and compared by independent samples *t*-test. To compare VO_2_ and VCO_2_, estimated means were calculated using ANCOVA after adjusting for lean mass as a covariate (common lean mass 23.6g). Data on VCO_2_/lean mass ratio and VO_2_/lean mass ratio are also presented as unadjusted means ± SEM and compared by independent samples *t*-test.

Due to the skewed distribution of several plasma and liver analytes, non-parametric methods were used for the plasma and liver biochemical measurements. These data are presented as median (25%, 75%), and compared by Mann-Whitney *U* test. The data obtained from histological scoring of livers was also analysed using Mann-Whitney *U* test.

PASW Statistics for Mac (20.0; SPSS Inc., Chicago, IL, USA) and GraphPad Prism (version 6.0f for Mac) were used for analysis and presentation of data. All tests were two-tailed and P <0.05 was considered significant.

## Results

### Body composition and liver fat

**Fig 1:**
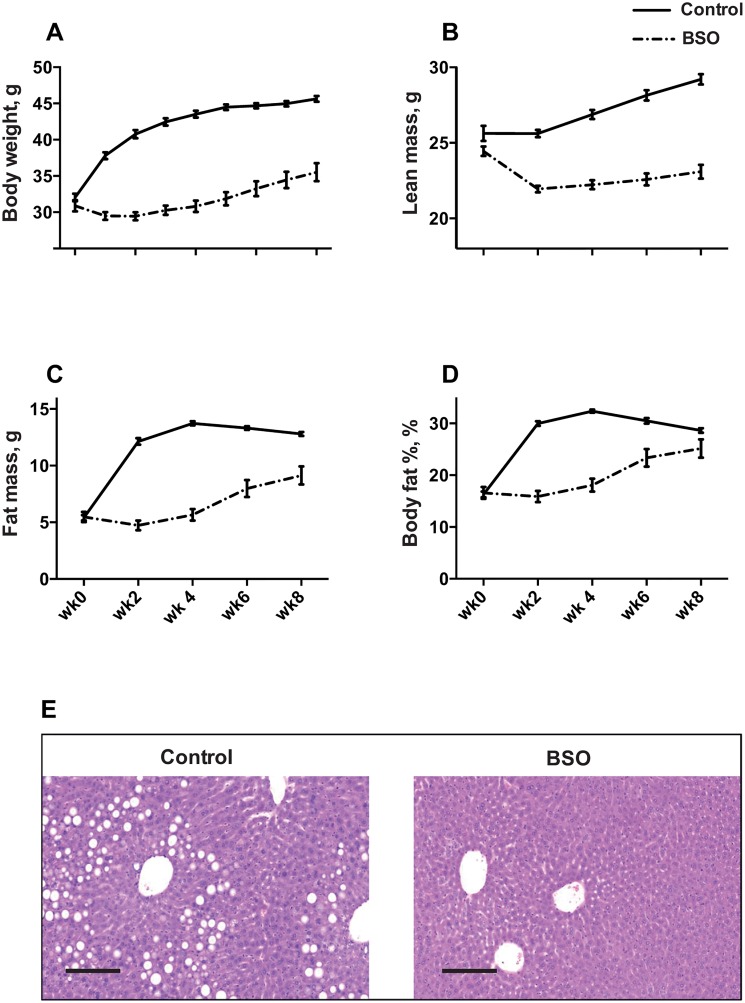
Effect of BSO on body composition. A-D Body weight and body composition in BSO-treated and control mice at the start of BSO treatment (wk-0) and every two weeks till termination 8 weeks later (wk-8). Data represents mean ± SEM from N = 20 (BSO) and N = 24 (controls). Depicted *P* values are from repeated measures ANOVA. E Photomicrograph of liver sections stained with H&E, showing hepatoceullar vacuolation in control mice and absence of vacuolation in BSO mice. Scale bar represents 300 μm.

Control C3H/HeH mice gained weight both from lean mass and fat mass on a HFD (Fig 1A–1C). The first 2 weeks on the HFD triggered the maximum increase in fat mass, with no growth in lean mass, which resulted in a near doubling of body fat% (from 16% to 30%, P<0.001; Fig 1D). This was followed by relatively stable fat mass from week 4 onwards. BSO decreased body weight, fat mass and lean mass (P<0.001 for all by repeated measures ANOVA). The maximum effect on fat mass was observed in the first 2 weeks on the HFD, where BSO completely prevented fat gain (Fig 1A). Mean terminal mesenteric fat mass was 0.21 g in BSO mice vs 0.32 g in controls (P = 0.001), but was not significantly different when normalized to total fat mass (i.e. no depot-specific reduction in mesenteric fat; data not shown).

On histopathological examination, large clear vacuoles (macrovesicular vacuolation) graded 3 (out of a maximum of 5), consistent with lipid accumulation, were present in all livers examined from control mice, while vacuolation was completely absent (P <0.001) in BSO-treated mice (Fig 1E). Liver weight, as a percent of total body weight, was not different in BSO-treated mice vs controls (P = 0.40, data not shown).

### Food intake, energy expenditure and locomotor activity

**Fig 2:**
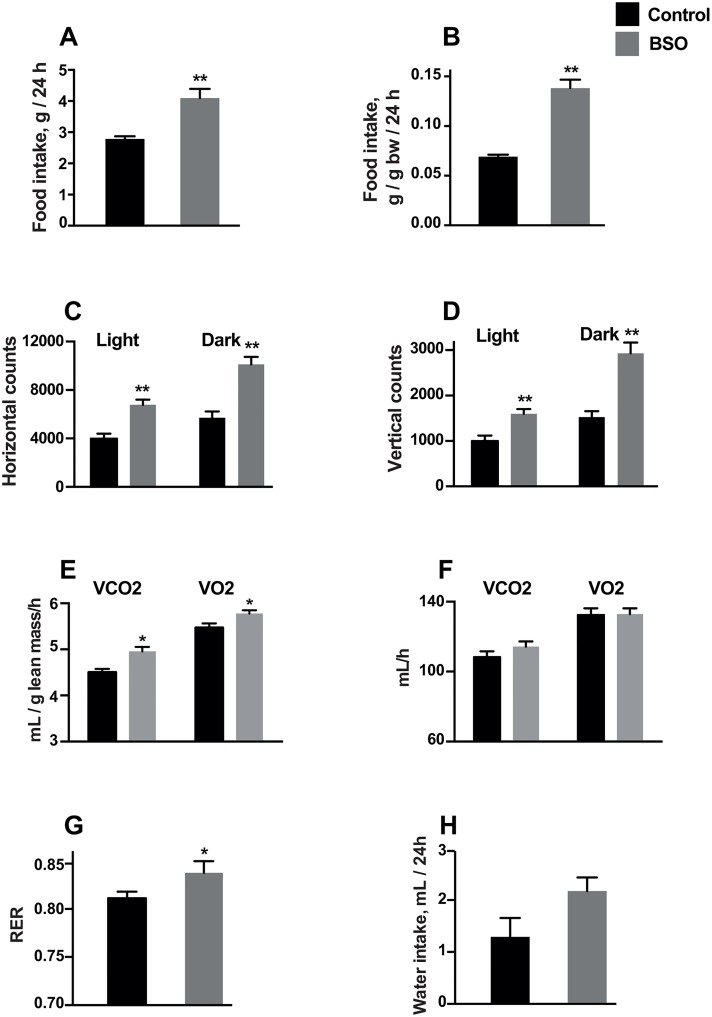
Phenomaster cage data at week 2 in BSO-treated and control mice. A, B: Food intake in g/day, and food intake normalized to body weight. C, D: Physical activity measured as infrared beam breaks in the horizontal and vertical planes during the light and dark phases of a 24-h period. E: O_2_ consumption (VO_2_)/lean mass ratio and CO_2_ production (VCO_2_)/ lean mass ratio. F: Adjusted mean VO_2_ and VCO_2_ controlling for lean mass as a covariate (at a common lean mass of 23.6 g) using general linear modelling. G: Respiratory exchange ratio (RER). H: Water intake in the studied groups. Data is presented as mean ± SEM and compared by independent samples T-test. N = 16–18 (control) and N = 17–20 (BSO). *, ** P<0.05 and P<0.001 respectively vs controls.

Food intake measured at week 2 was higher in BSO-treated mice (Fig 2A and 2B). BSO also enhanced physical activity, particularly during the dark phase (Fig 2C and 2D). We used two methods to normalize energy-expenditure related data for lean mass. Using the ratio method, VO_2_/lean mass ratio and VCO_2_/lean mass ratio were significantly higher in BSO-treated mice (Fig 2E). However, mean VO_2_ and VCO_2_, adjusted for lean mass as a covariate, as recommended [28], were not significantly different in BSO vs control mice (Fig 2F). BSO-treated mice had a higher RER, reflecting a shift towards carbohydrate oxidation. There was no significant effect of BSO on water intake (Fig 2H).

### Plasma biochemistry and peripheral insulin sensitivity

**Fig 3:**
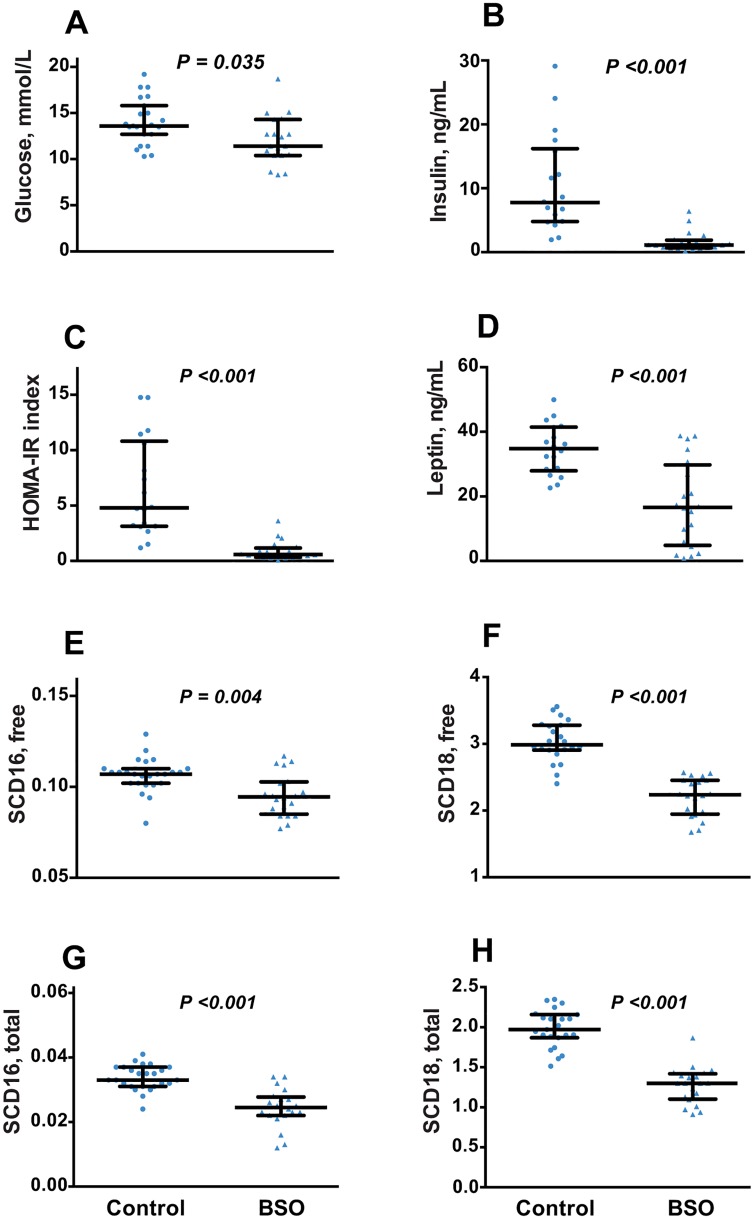
Insulin sensitivity and SCD activity in BSO-treated and control mice. A, B: Plasma glucose and insulin measured after a 6h fast after 6 weeks of BSO treatment. C: Homeostatic model of insulin resistance index (HOMA-IR) calculated from the plasma glucose and insulin values. D: Plasma leptin after 6 weeks of BSO treatment. E-H: Stearoyl coenzyme A desaturase (SCD-16 and SCD-18) activity indices calculated from plasma free fatty acid profile (E, F) and from fatty acid profile in total plasma lipids (G, H) after 8 weeks of BSO treatment. Data is presented as median, 25^th^ and 75^th^ percentiles, with individual data plotted; N = 20 (BSO) and N = 18 (controls). Groups were compared by Mann-Whitney *U* test.

Compared to control, BSO induced a 12.5% reduction in fasting plasma glucose at week 6 of treatment (P = 0.035), with a marked decrease in insulinemia, and hence HOMA-IR (Fig 3A–3C). Plasma glucose measured in overnight-fasted plasma at termination (week 8) was, however, not significantly lower in BSO-treated mice (Table 1).

**Table 1:** Plasma clinical biochemistry in BSO-treated mice after 8 weeks^1^.

	Control (N = 23)	BSO (N = 20)	*BSO/Control* ^2^ *(%)*
Glucose, mmol/L	11.3 (10.1, 12.3)	10.1 (8.08, 13.1)	*89*
Triglycerides, mmol/L	2.84 (2.51, 3.09)	1.78 (1.26, 1.91)	63**
Total cholesterol, mmol/L	5.1 (4.79, 5.44)	5.05 (4.68, 5.36)	*99*
LDL-cholesterol, mmol/L	0.99 (0.93, 1.06)	0.94 (0.86, 1.05)	*95*
HDL-cholesterol, mmol/L	3.51 (3.29, 3.73)	3.61 (3.44, 3.83)	*103*
Albumin, g/L	26.8 (26.4, 27.7)	24.4 (23.7, 25.5)	91**
Total protein, g/L	52.8 (52.0, 54.3)	48.7 (48.0, 50.6)	92**
ALT, U/L	34 (28, 42)	22 (18.5, 27.5)	65**
Creatinine, μmol/L	10.2 (9.7, 11.7)	8.9 (8.3, 9.4)	87**

^1^. Data represents median (25%, 75%). Parameters were measured after an overnight fast.

**P<0.001, Mann Whitney *U* test.

ALT, alanine aminotransferase.

^2^. Calculated using medians.

Plasma leptin was also decreased (Fig 3D). Since leptin is produced in proportion to fat mass [29], we calculated the fat-mass adjusted leptin plasma concentration. The median (25^th^ %, 75^th^ %) values were 2.69 (2.12, 3.00) ng/mL/kg fat mass in controls, compared to 1.69 (1.23, 2.69) ng/mL/kg fat mass in BSO-treated mice (P <0.001). Thus the decreased leptin in BSO-treated mice was greater than could be explained by reduction in fat mass alone, indicating a specific effect of BSO in decreasing leptin production.

Other fasting plasma clinical biochemistry measurements are shown in Table 1. A marked decrease in plasma triglycerides at termination (week 8) was observed (median 2.84 mmol/L in controls vs 1.78 mmol/L in BSO-treated mice, P <0.001), but BSO did not alter total, HDL- or LDL-cholesterol. Modest, but significant decreases in plasma total protein and albumin (8% and 9%, respectively, P <0.001 for both) were observed in BSO-treated mice versus controls. Plasma ALT and creatinine results suggested that hepatic and renal functions were not adversely affected by BSO treatment. Plasma GGT activity was below the limit of detection in both BSO-treated mice and controls.

### Plasma fatty acid profile

#### Free fatty acids

Plasma total and free concentrations of palmitic, palmitoleic, stearic and oleic acids are shown in Table 2, while the remaining fatty acids measured are reported in S1 Table in the Supporting Information. Most plasma free fatty acids were decreased by BSO relative to controls, with the greatest decrease observed in dihomo-γ-linolenic acid (44% lower), as well as in monounsaturated products of SCD, namely oleic and palmitoleic acids (P<0.001 for all). As a result, SCD-16 (P = 0.004) and SCD-18 (P <0.001) activity indices at week 8 were decreased in BSO-treated mice relative to controls, with SCD-18 showing the greatest effect (25% lower, P <0.001; Fig 3E and 3F). Plasma free fatty acid concentration, calculated as the sum of all individual fatty acids measured (S1 Table), was 21% lower in BSO-treated mice relative to controls (P <0.001; Table 2).

**Table 2:** Effect of BSO on fatty acid precursors and products of stearoyl coenzyme A desaturase ^1^.

	Control (N = 23)	BSO (N = 20)	*BSO/ Control*^2^ *(%)*
***Concentration in total plasma lipids***			
C16:0 (palmitic)	3506 (3394, 3622)	3019 (2801, 3152)	86**
C16:1n-7 (palmitoleic)	118 (107, 133)	73.5 (59.8, 85.1)	62**
C18:0 (stearic)	1284 (1238, 1386)	1267 (1170, 1341)	*99*
C18:1n9 (oleic)	2581 (2310, 2892)	1631 (1389, 1863)	63**
***Free fatty acid concentration***			
C16:0 (palmitic)	513 (447, 531)	405 (366, 470)	79*
C16:1n-7 (palmitoleic)	53.7 (45.0, 58.8)	37.1 (32.5, 44.7)	69**
C18:0 (stearic)	133 (116, 153)	132 (122, 173)	*99*
C18:1n9 (oleic)	417 (343, 443)	305 (285, 354)	73**
Sum of free fatty acids^3^	1554 (1379, 1670)	1230 (1094, 1450)	79**

^1^. Data represents median (25%, 75%). All concentrations are in μmol/L plasma, and are measured after an overnight fast.

*P<0.05;

**P<0.001, Mann Whitney *U* test. Stearoyl coenzyme A desaturase converts palmitic to palmitoleic, and stearic to oleic acids.

^2^. Calculated using medians.

^3^. Sum of all the free fatty acids reported in S1 Table.

#### Fatty acid profile in total plasma lipids

Total plasma concentrations of several fatty acids were lower in BSO-treated mice, particularly dihomo-γ-linolenic acid, oleic and palmitoleic acids (37–40% lower, P<0.001 for all; Table 2 and S1 Table), which were the ones maximally decreased in the free fatty acid profile. SCD-16 and SCD-18 activity indices calculated in total plasma lipids were 27% and 34% respectively lower in BSO-treated mice at termination compared to controls (P <0.001 for both; Fig 3G and 3H). BSO had no effect on total stearic or γ-linolenic acid concentrations.

### Thiol profile in plasma and liver

**Fig 4:**
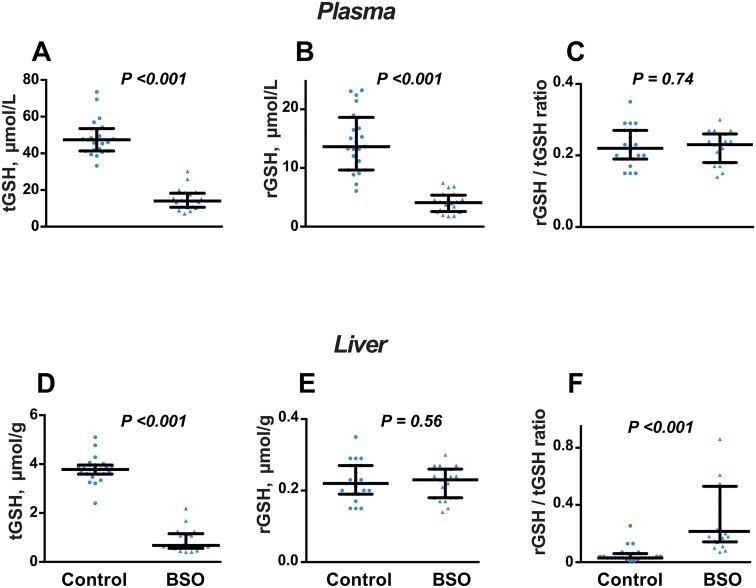
Effect of BSO on glutathione status after 8 weeks of treatment. A-C: Plasma total glutathione (tGSH), reduced glutathione (rGSH) and rGSH/tGSH ratio. D-F: Liver tGSH, rGSH and rGSH/tGSH ratio. Data is presented as median, 25^th^ and 75^th^ percentiles, with individual data plotted; N = 20 (BSO) and N = 23 (controls). Groups were compared by Mann-Whitney *U* test.

Total and reduced GSH in plasma were decreased by BSO treatment (P <0.001 for both), but the rGSH/tGSH ratio was unchanged (Fig 4A–4C). In liver, rGSH was not altered by BSO treatment, while tGSH was decreased, so the rGSH/tGSH ratio was higher in BSO-treated mice compared to controls (P <0.001; Fig 4D–4F).

**Fig 5:**
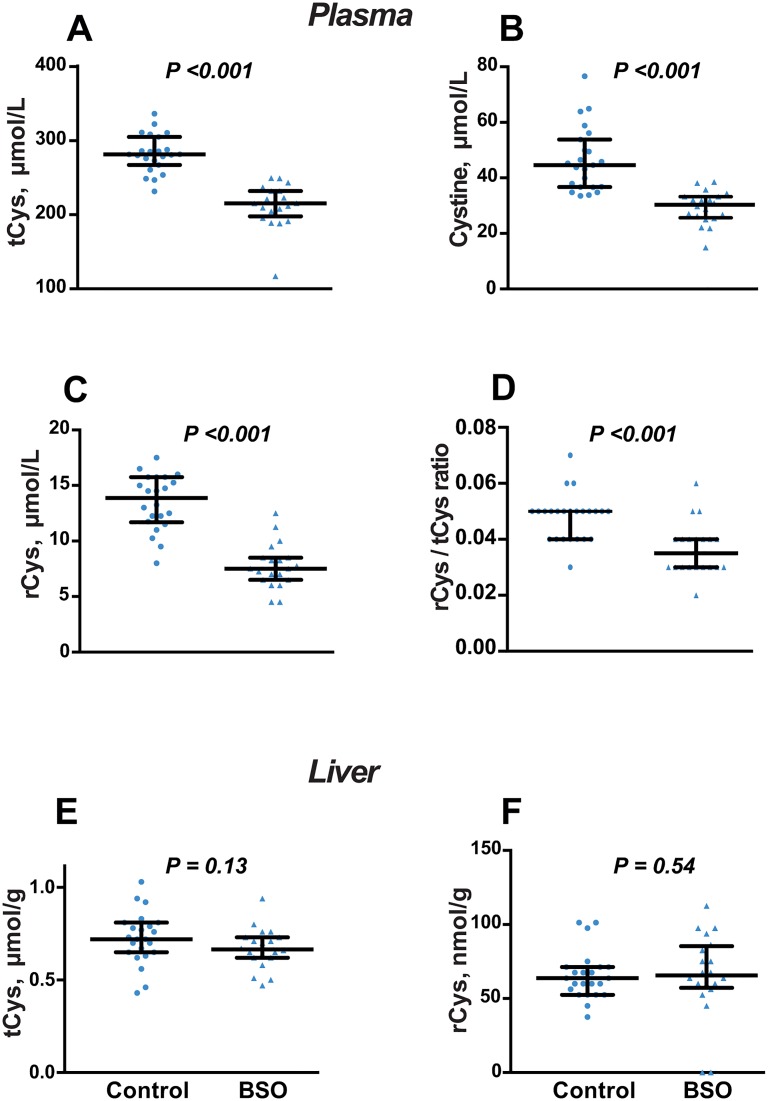
Effect of BSO on cysteine after 8 weeks of treatment. A-D: Plasma total cysteine (tCys), reduced cysteine (rCys), cystine, and rCys/tCys ratio. E, F: Liver tCys and rCys. Data is presented as median, 25^th^ and 75^th^ percentiles, with individual data plotted; N = 20 (BSO) and N = 23 (controls). Groups were compared by Mann-Whitney *U* test.

All cysteine forms were decreased in plasma (Fig 5A–5C), with a proportionally greater decrease in rCys, such that the rCys/tCys ratio was decreased (Fig 5D). Similarly, the plasma rHcy/tHcy ratio was decreased by 67%, due to an absolute increase in tHcy and a decrease in rHcy (Table 1). BSO had no effect on liver concentrations of total or reduced cysteine (Fig 5E and 5F) or the rCys/tCys ratio in liver (not shown).

### Amino acid profile in plasma and liver

The effect of BSO on amino acid concentrations was not consistent across plasma and liver, with several amino acids showing contrasting changes in both compartments (Table 3). For example, cystathionine, ornithine, as well as branched chain and aromatic amino acids were significantly increased in liver (by 12–38%), but decreased or unchanged in plasma. Glutamic acid, a substrate of the inhibited GCL enzyme, appeared to accumulate both in plasma (19% higher; P = 0.06 in BSO-treated micevs control), and liver. The quantitatively greatest effect of BSO was on plasma tHcy, which was 66% higher in BSO-treated mice compared to controls (P<0.001), and conversely, was ~30% lower in liver. In concert with the decreased cysteine in plasma, its product taurine was significantly decreased in plasma and liver of BSO-treated mice (Table 3).

**Table 3:** Effect of BSO on amino acid profile and related variables ^1^.

	Plasma		*BSO/ Control*^2^ *(%)*	Liver		*BSO/ Control (%)*
	Control (N = 23)	BSO (N = 20)		Control (N = 23)	BSO (N = 20)	
***Sulfur amino acids and related metabolites***						
Methionine	55.1 (51.9, 61.1)	58.9 (55.7, 64.5)	*107*	1.51 (1.39, 1.68)	1.80 (1.66, 2.24)	119**
SAM	301 (274, 331)	248 (227, 262)	82**	2.03 (1.73, 2.10)	1.90 (1.7, 2.18)	*94*
SAH	58.1 (42.2, 73.6)	47.3 (33.4, 60.3)	*81*	11.3 (9.9, 12.8)	14.0 (11.2, 16.1)	123*
SAM/ SAH	5.01 (4.73, 6.70)	5.12 (3.71, 6.09)	*102*	0.17 (0.15, 0.19)	0.14 (0.11, 0.19)	82*
tHcy	4.70 (4.30, 5.0)	7.80 (7.2, 9.5)	166**	11.7 (9.4, 13.5)	8.35 (7.90, 12.1)	71*
rHcy	170 (150, 230)	130 (110, 155)	76**	ND^3^	ND	
Cystathionine	845 (771, 1009)	670 (608, 755)	79**	1.44 (1.35, 1.61)	1.98 (1.65, 2.2)	138**
Taurine	344 (328, 373)	263 (236, 288)	76**	15.4 (14.9, 16.3)	12.6 (11.5, 13.2)	82**
***Branched-chain and aromatic amino acids***						
Leucine	239 (230, 258)	218 (177, 227)	91**	6.93 (6.16, 7.70)	7.77 (7.40, 10.09)	112*
Isoleucine	139 (126, 155)	122 (105, 136)	88*	2.84 (2.63, 3.22)	3.50 (3.11, 4.13)	123**
Valine	309 (279, 322)	274 (225, 295)	89*	5.29 (4.87, 5.71)	5.96 (5.48, 7.13)	113*
Phenylalanine	122 (110, 130)	114 (108, 122)	*93*	5.15 (4.47, 5.58)	5.79 (5.57, 7.67)	112**
Tyrosine	81.4 (72.8, 91.9)	94.7 (80.2, 105.3)	*116*	1.80 (1.49, 2.27)	2.37 (1.67, 2.82)	132*
Tryptophan	122 (110, 138)	103 (94, 116)	84**	0.59 (0.48, 0.65)	0.66 (0.60, 0.83)	112*
***Others***						
Glutamine	812 (784, 871)	857 (824, 925)	106*	4.39 (3.82, 5.33)	5.71 (4.95, 7.85)	130*
Glutamic acid	76.8 (67.2, 87.2)	91.5 (74.0, 125.5)	*119*^4^	5.50 (4.75, 6.53)	6.57 (5.70, 7.77)	119*
Serine	239 (216, 257)	261 (230, 302)	109*	13.8 (12.8, 14.7)	15.1 (14.2, 17.7)	110*
Proline	80.5 (69.7, 91.3)	91.9 (81.7, 107.0)	114*	2.48 (2.17, 2.91)	2.98 (2.64, 3.97)	120*
Ornithine	93 (81, 118)	72.8 (67.3, 84.8)	78**	3.84 (3.47, 4.50)	4.64 (4.05, 6.11)	121*

^1^ Data represents median (25%, 75%). All plasma and liver analytes are in μmol/L and μmol/g respectively, except for SAM, SAH, cystathionine and rHcy (nmol/L and nmol/g in plasma and liver respectively). rHcy, reduced homocysteine, SAM, S-adenosylmethionine; SAH, S-adenosylhomocysteine, tHcy, total homocysteine.

*P<0.05;

**P<0.001, Mann Whitney *U* test.

^2^ Calculated using medians.

^3^ ND, not determined.

^4^ P = 0.06.

### Urine glutathione and sulfur amino acid excretion

**Fig 6:**
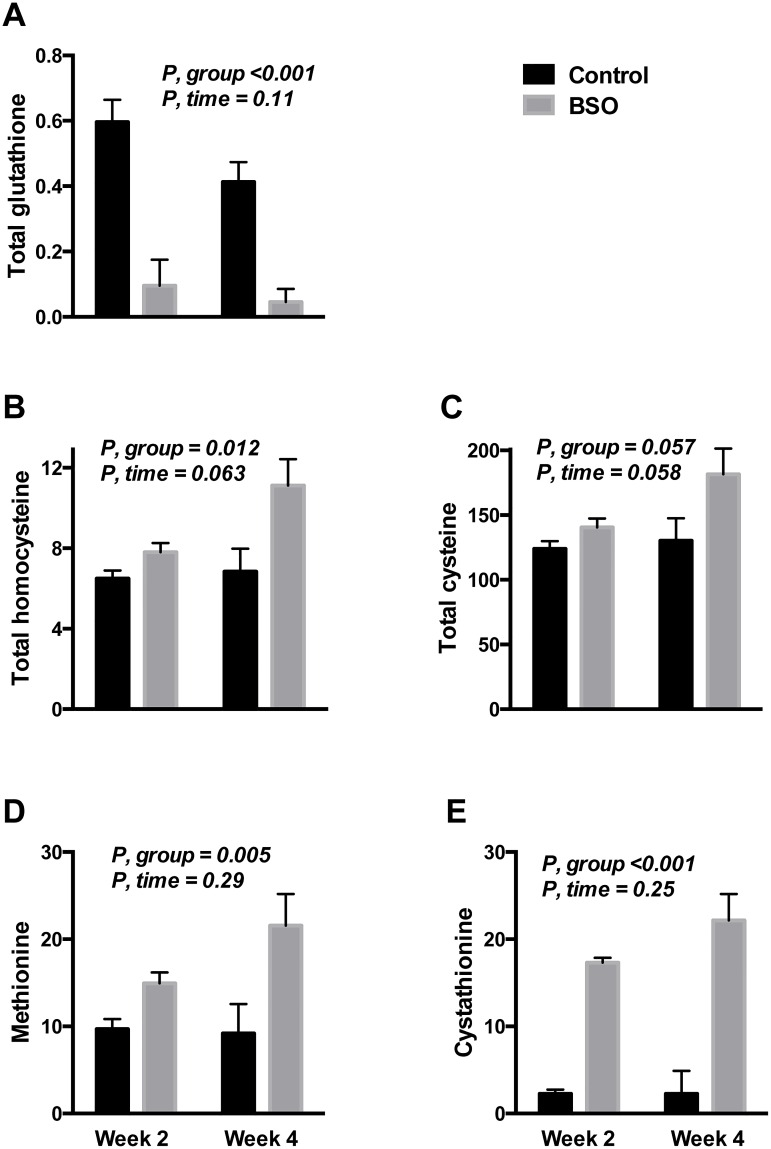
Effect of BSO on urine excretion of glutathione and upstream sulfur amino acids. A-E: Urine concentration of total glutathione, methionine, total homocysteine, cystathionine and total cysteine, in nmol/μmol creatinine during the 2^nd^ and 4^th^ weeks of BSO treatment. Depicted P values are from repeated measures ANOVA. N = 6 BSO-treated mice, and 8 controls. Data is from a different cohort of mice of the same age and sex.

In an attempt to explain the decreased plasma tCys despite the block of cysteine utilization in glutathione synthesis, we determined the urine concentration of tCys, tGSH and upstream sulfur amino acids after 2 and 4 weeks of BSO treatment. tGSH in urine from BSO-treated mice was decreased (P <0.001) to under 20% of controls at both time-points (Fig 6A). In contrast, tCys excretion appeared to increase (P = 0.057) by 5% and 29% at week 2 and 4 respectively (P = 0.058 for the effect of time by repeated measures ANOVA; Fig 6C). The increase in tCys excretion appeared to track urine tHcy excretion (Fig 6B). BSO treatment also triggered an increase in urine methionine concentration, as well as a several-fold rise in urine cystathionine (Fig 6D and 6E).

## Discussion

We conducted comprehensive phenotypic and biochemical characterization of BSO-treated mice that are resistant to HFD-induced obesity and insulin resistance [13]. Block of GSH synthesis decreased all forms of plasma cysteine, an amino acid positively associated with adiposity, SCD activity and insulin resistance in rodents and humans [5, 15, 16, 30, 31]. BSO decreased plasma free fatty acids and raised RER, suggesting suppressed lipolysis and higher carbohydrate utilization. Other novel findings include protection against fatty liver vacuolation, and large reductions in plasma total and free oleic and palmitoleic acids, suggesting suppression of SCD [32] and of hepatic lipogenesis [33]. Our findings provide insight into some factors contributing to resistance to obesity and obesity-related morbidity in response to BSO treatment.

### Effect of BSO on metabolic phenotypes

Locomotor activity was increased by BSO, as previously noted [13]. Physical activity shows strong inverse correlations with weight gain in mice [34]. However, behavioural studies in *Gclm*^*-/-*^ mice show that GSH depletion triggers hyper-locomotion that selectively occurs in novel and mildly stressful environments but not in the home cage [35]. Since the mice were individually housed in unfamiliar cages to assess physical activity, their hyperactivity may reflect a transient response to cage novelty rather than a lasting phenotype. The hyperphagia may also have a similar explanation, given that others did not note an effect of BSO on food intake [13, 36].

Our data using large group sizes suggests that energy expenditure is not significantly increased by BSO, when analysed by the recommended ANCOVA method [28]. Although higher energy expenditure was reported in BSO-treated mice, it was presented as a ratio to body weight [13], which may artificially inflate the differences [28]. The decreased weight gain in BSO-treated mice may alternatively result from an effect on nutrient absorption, or by subtle shifts in mitochondrial function, or fat metabolism [37], that are not linked to measurable effects on energy expenditure.

The insulin resistance may predispose to fatty liver via impairment of insulin-suppression of lipolysis, increasing free fatty acid flux towards the liver [38]. The increased insulin sensitivity and decreased free fatty acids observed in response to BSO may partly explain the absence of fatty liver in BSO-treated mice. Substantial down-regulation of several hepatic lipogenic enzymes and transcription factors was also reported following BSO administration in rats [37], and in *Gclm-/-* mice [12]. In the present study we also observed a marked reduction in plasma palmitoleic acid, a fatty acid product of SCD that is a strong indicator of hepatic lipogenesis [33]. Interestingly, plasma free and total dihomo- γ-linolenic acid, a fatty acid that is consistently associated with human obesity [39], was also markedly decreased in plasma.

### Effect of BSO on cysteine and glutathione

The two key factors determining GSH synthesis are cysteine availability and GCL activity [40], so the decreased plasma tCys following block of GSH synthesis mice is unexpected. Unlike tCys, glutamate, another precursor of GSH, accumulated in plasma and liver in BSO-treated mice. Kendig et al similarly observed that plasma rCys and cystine decrease in *Gclm-/-* mice [11]. They reported increased plasma insulin and suggested that the role of insulin in regulating cystathionine beta synthase (CBS) [41] may have contributed to the thiol changes. We did not observe similar increases in insulin in response to BSO; in fact insulin levels were >6-fold lower. Another distinct finding in our BSO-treated mice was that, while tGSH was markedly decreased in liver and plasma, rGSH was maintained in liver. Liver rGSH is critical for normal insulin sensitivity [42].

It is difficult to explain the paradoxical decrease in plasma tCys. Our observation that liver tCys was unaffected by BSO suggests that the drop in plasma tCys was due to a factor that selectively influences circulating levels. Three such factors can be postulated. The first is that plasma GGT activity, which ultimately releases cysteine extracellularly, was decreased due to depletion of the substrate, GSH [19]. In line with this, GGT null mice feature low plasma cysteine [43]. However, GGT activity was undetectable in both the BSO and control groups, so it was not possible to confirm or refute this hypothesis. An alternative explanation is linked to the observed increase in plasma tHcy. Hyperhomocysteinemia is associated with displacement of cysteine from protein-binding sites [44], and increased excretion of homocysteine-cysteine mixed disulfides [45]. We observed a progressive increase in urine excretion of both tCys and tHcy in BSO exposed mice. The non-thiol sulfur amino acids methionine and cystathionine were also increased in urine, suggesting loss of cysteine precursors, possibly related to the effect of BSO in kidney. Further, the increased liver glutamate in BSO mice may stimulate the glutamate cystine antiporter [46], leading to increased export of glutamate from cells in exchange for cystine from plasma. This is consistent with the changes observed in both plasma and liver. Thus, the lowering of plasma tCys is probably related to a number of secondary effects of BSO on sulfur amino acid metabolism, transport and excretion. That liver tCys was maintained in BSO mice despite loss of cysteine precursors in urine suggests prioritization of maintaining liver cysteine, at least partly via inhibiting its catabolism to taurine [47], as evidenced by the low taurine in liver and plasma.

### Dissecting the effects of cysteine and glutathione depletion on the lean phenotype

Similar fat loss, with low plasma tCys and tGSH, and SCD inhibition as occurs in response to BSO, have been observed in several dietary and genetic manipulations of the sulfur amino acid pathway [18]. These include methionine restriction and homozygous CBS deletion. In both models, fat gain and hepatic SCD expression were rescued by therapy that restores plasma tCys [30, 48]. In CBS-/- mice, therapy that normalised GSH status, but not plasma tCys, failed to restore fat gain [49]. We therefore postulate that the lean phenotype in BSO-treated mice may be linked to their low circulating cysteine.

SCD and several other lipogenic enzymes are under regulation by the transcription factor PPAR-γ [50]. PPAR-γ was markedly upregulated in liver and adipose tissue of mice fed a high-cystine diet [51]. In preadipocytes, low cysteine inhibited PPAR-γ expression and adipocyte differentiation in a dose-dependent manner [52]. Recently, CDO, an enzyme that is strongly induced by cysteine [47], was shown to promote adipogenesis via activating PPAR-γ [53]. Taurine was decreased in BSO-treated mice, suggesting down-regulation of CDO. The possibility of CDO suppression in BSO-treated mice warrants investigation as a potential factor in their decreased adiposity.

### BSO phenotype and toxicity

Because the metabolic benefits of BSO challenge existing evidence that GSH depletion is detrimental to health, it has been suggested that the leanness of BSO-treated mice results from toxicity due to the relatively high dose [54]. BSO has indeed been reported to decrease glutathione peroxidase activity as well as other antioxidant enzymes that do not use GSH as a substrate (e.g. catalase), denoting compromised antioxidant status [55]. In the present study, however, the ratio of reduced to total GSH was maintained in plasma and increased in liver. Serum creatinine and ALT after 56 days of BSO treatment were lower than HFD-fed controls, suggesting that neither renal nor liver function were impaired. Yet only the liver was examined microscopically so the possibility of subclinical renal pathology cannot be excluded. The decrease in creatinine probably reflects the decreased lean mass, which is the major source of the creatinine precursor, creatine. The decrease in ALT appeared to parallel a general decrease in proteins synthesized by the liver. Plasma albumin and total protein decreased, despite a significant increase in liver concentration of all proteinogenic amino acids.

### Summary

In summary, we attempted to extend current data on the obesity-resistance conferred by GSH depletion [13, 14] by characterizing the metabolomic profile of BSO-treated mice. Inhibition of GSH synthesis had widespread effects on the amino acid and fatty acid metabolomes, and on protein and fat metabolism. Our findings raise the possibility that the leanness of BSO-treated mice may be linked to their low concentrations of circulating cysteine. This hypothesis can be tested in future work by investigating whether cysteine supplementation of BSO-treated mice can rescue fat gain.

## Ethical Approval

All experimental procedures were conducted according to the Animal (Scientific Procedures) Act 1986 Amendment Regulations 2012 (SI 4 2012/3039), and in accordance with UK Home Office welfare guidelines and project license restrictions.

## Supporting Information

S1 Fig The sulfur amino acid metabolic pathway and site of action of BSO. Methionine is the precursor of the universal methyl-donor SAM. Upon donating its methyl group, SAM is converted to SAH, and hence, homocysteine. Homocysteine is either remethylated to methionine or undergoes transsulfuration with production of cystathionine and subsequently, cysteine. GCL is the rate-limiting enzyme in synthesis of glutathione from cysteine, and is the enzyme inhibited by BSO. Located at cell membranes, GGT catalyzes breakdown of glutathione to glutamate and cysteinylglycine, which ultimately releases cysteine, in the **γ**-glutamyl cycle. Cysteine is also the precursor of taurine, in which the cysteine dioxygenase (CDO) reaction is the first irreversible step. For clarity, not all pathways, co-substrates or products are shown and some intermediates are omitted (indicated by dotted lines). CBS, cystathionine beta synthase; CDO, cysteine dioxygenase; CS, cysteinesulfinic acid; CSE, cystathionine gamma lyase; GGCS, gamma glutamylcysteine synthase; GGT, **γ**-glutamyl transferase; SAH, S-adenosylhomocysteine; SAM, S-adenosylmethionine.
(EPS)

S1 Table Effect of BSO on plasma fatty acid profile. (DOCX)
