# Supplementary material for: Exploring the Lean Phenotype of Glutathione-Depleted Mice: Thiol, Amino Acid and Fatty Acid Profiles
Source: PLoS One. 2016 Oct 27;11(10):e0163214. doi: 10.1371/journal.pone.0163214 (PMC5082875; doi:10.1371/journal.pone.0163214)
Supplement: S1 Table — (DOCX) [file pone.0163214.s002.docx]

### S1 Table. Effect of BSO on plasma fatty acid profile ^1^

|  | **Control (N = 23)** | **BSO (N = 20)** | ***BSO/ Control (%)^2^*** |
| --- | --- | --- | --- |
| ***Fatty acid profile in total plasma lipids*** | | | |
| C12:0 (lauric) | 2.68 (2.30, 2.86) | 2.56 (2.12, 3.20) | *96* |
| C14:0 (myristic) | 22.1 (20.1, 24.6) | 18.2 (15.9, 20.5) | *82** |
| C16:0 (palmitic) | 3506 (3394, 3622) | 3019 (2801, 3152) | *86*** |
| C16:1n-7 (palmitoleic) | 118 (107, 133) | 73.5 (59.8, 85.1) | *62*** |
| C18:0 (stearic) | 1284 (1238, 1386) | 1267 (1170, 1341) | *99* |
| C18:1n9 (oleic) | 2581 (2310, 2892) | 1631 (1389, 1863) | *63*** |
| C18:2n6 (linoleic) | 3292 (3073, 3539) | 3025 (2802, 3277) | *92** |
| C18:3n6 (α-linolenic) | 63.2 (48.7, 68.4) | 53.3 (37.8, 61.5) | *84* |
| C18:3n3 (γ-linolenic) | 56.3 (49.0, 66.7) | 59.3 (52.4, 68.4) | *105* |
| C20:3n6 (dihomo- γ-linolenic) | 168 (151, 181) | 100 (86, 116) | *60*** |
| C20:4n6 (arachidonic) | 4453 (4323, 4832) | 4080 (3711, 4279) | *92** |
| C20:5n3 ( eicosapentaenoic) | 1597 (1519, 1694) | 1279 (1118, 1415) | *80*** |
| C22:6n3 ( docosahexaenoic ) | 59.2 (49.3, 63.7) | 47.2 (39.7, 57.0) | *80** |
| ***Free fatty acids*** | | | |
| C14:0 (myristic) | 7.61 (6.98, 8.30) | 7.14 (6.47, 8.33) | *93* |
| C16:0 (palmitic) | 513 (447, 531) | 405 (366, 470) | *79** |
| C16:1n-7 (palmitoleic) | 53.7 (45.0, 58.8) | 37.1 (32.5, 44.7) | *69*** |
| C18:0 (stearic) | 133 (116, 153) | 132 (122, 173) | *99* |
| C18:1n9 (oleic) | 417 (343, 443) | 305 (285, 354) | *73*** |
| C18:2n6 (linoleic) | 325 (295, 349) | 277 (239, 298) | *85*** |
| C20:3n6 (dihomo- γ-linolenic) | 11.2 (9.3, 12.1) | 6.61 (5.67, 7.98) | *59*** |
| C20:4n6 (arachidonic) | 87.8 (71.2, 103.3) | 77.8 (66.5, 99.7) | *89* |
| Sum of free fatty acids | 1554 (1379, 1670) | 1230 (1094, 1450) | *79*** |

^1^. Data represents median (25%, 75%). All concentrations are in μmol/L, and measured after an overnight fast. *P<0.05; **P<0.001, Mann Whitney *U* test.

^2^. Calculated using medians.
